# Infective Endocarditis in an Intravenous Drug User: Clinical Challenges, Ethical Dilemmas, and Multidisciplinary Management

**DOI:** 10.7759/cureus.86683

**Published:** 2025-06-24

**Authors:** Munna Hazime, Shailes Paudel, Alexander A Restum, Wisam Mnkana

**Affiliations:** 1 Medical Education, Wayne State University School of Medicine, Detroit, USA; 2 Internal Medicine, Corewell Health Dearborn Hospital, Dearborn, USA

**Keywords:** cardiology research, infective endocarditis, intravenous drug user, multidisciplinary management, multi-modality pain management

## Abstract

Infective endocarditis (IE), an infection of the endocardial surface of the heart, is a severe condition frequently seen in intravenous drug users (IVDU), primarily driven by *Staphylococcus aureus*. This case study examines the intricate clinical, ethical, and multidisciplinary challenges in managing a 48-year-old female with a history of IVDU, who presented with IE and multisystem complications. Initial evaluation revealed systemic septicemia with disseminated intravascular coagulation, septic emboli, and significant tricuspid valve involvement. Comprehensive management included tailored antibiotic therapy, infectious disease consultation, and palliative care integration, complicated by newly diagnosed HIV, emotional distress, and chronic pain.

This report highlights the unique pathophysiology of IE in IVDU, driven by endothelial trauma and microbial colonization, often affecting the tricuspid valve. Ethical dilemmas arose when the patient, fully informed of her prognosis, elected to discontinue treatment and transition to hospice care, creating tension between respecting autonomy and ensuring beneficence. These challenges necessitated multidisciplinary collaboration to balance medical interventions with patient preferences. The case underscores the critical importance of prompt diagnosis, ethical sensitivity, and patient-centered care in managing IE in high-risk populations. It advocates for compassionate communication and respect for patient autonomy while navigating complex clinical and ethical decisions.

## Introduction

Infective endocarditis (IE) is an infection of the endocardial layer of the heart. It commonly arises on heart valves and is a progressive process that demands timely recognition and intervention. It is caused by bacteria, most typically *Staphylococcus aureus*, *viridans* streptococci, enterococci, and coagulase-negative staphylococci. Risk factors for IE include prosthetic heart valves, structural heart disease, intravenous drug use (IVDU), and invasive procedures. Our patient’s etiology was related to a history of heroin IVDU. The incidence of IE in IVDU is approximately 2% to 5% per year [1].

In this case report, we present an in-depth analysis of IE in an IV drug user, emphasizing the critical role of prompt diagnosis and collaborative care. It explores the clinical presentation, diagnostic approaches, and management strategies, highlighting the importance of swift recognition and intervention to address this serious complication in a high-risk patient group. Additionally, the patient’s complex history raised several ethical dilemmas, which will also be explored in this discussion.

## Case presentation

A 48-year-old female with a significant past medical history of IVDU, heroin dependency (last use four years ago), methicillin-resistant *Staphylococcus aureus* (MRSA) septicemia, methicillin-sensitive *Staphylococcus aureus *(MSSA) bacteremia, septic emboli, chronic obstructive pulmonary disease (COPD), angina, anemia, chronic back pain (secondary to a fall from 13 feet at age 18), and cholelithiasis presented to the emergency department with complaints of back pain, substernal chest pain radiating to the left, and shortness of breath for the past week. EMS noted an oxygen saturation of 88% on room air, improving to 94% on 2L nasal cannula. Initial evaluation revealed tachypnea (RR 48), tachycardia (HR 100), hypotension (BP 96/54), and an ill-appearing patient with scleral icterus, jaundice, and IV injection marks. Laboratory findings showed hyponatremia; elevated creatinine, procalcitonin, lactic acid, and bilirubin; thrombocytopenia; and coagulopathy suggestive of disseminated intravascular coagulation (DIC). Blood cultures grew MSSA. Chest X-ray revealed multifocal patchy nodular opacities consistent with septic emboli, biapical blebs, degenerative lumbar spine changes, cholelithiasis with gallbladder wall thickening, and hepatosplenomegaly. The patient was started on broad-spectrum antibiotics (vancomycin and ceftriaxone), IV fluids, oxygen therapy, and COPD management. Evaluation for infective endocarditis was initiated.

Management

**Figure 1:**
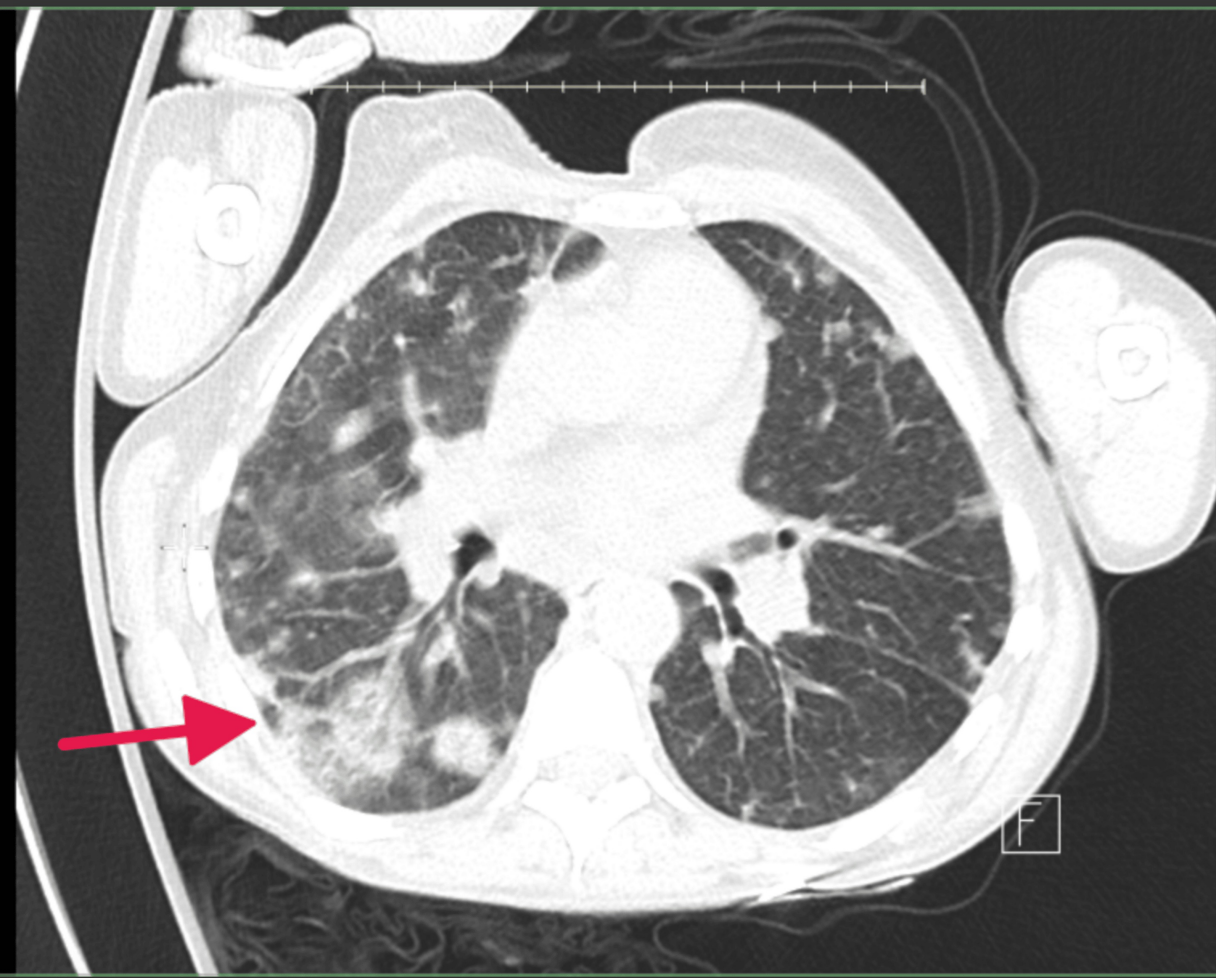
CT angiography of the chest and abdomen: bilateral septic emboli at the time of admission.

**Figure 2:**
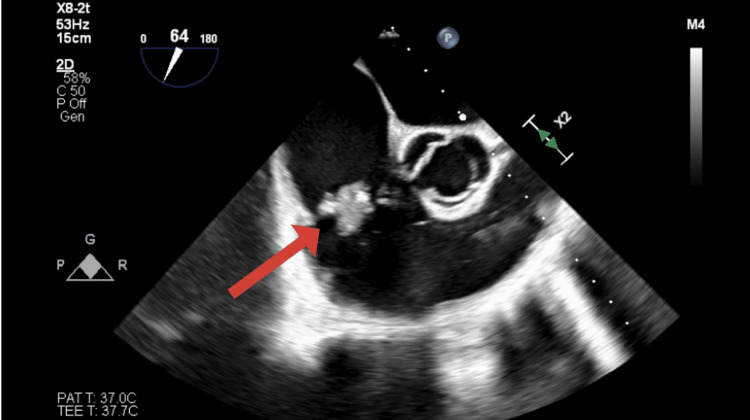
Transesophageal echocardiogram (TEE) of the tricuspid valve. Transesophageal echocardiogram (TEE) showing a large vegetation on the tricuspid valve, consistent with infective endocarditis in the setting of intravenous drug use (IVDU).

The patient's initial management included broad-spectrum antibiotics (vancomycin 25 mg/kg and ceftriaxone 2 g IV), later narrowed to cefazolin based on culture susceptibilities. Due to continued septicemia and persistent positive blood cultures, therapy was transitioned to nafcillin. Blood cultures turned negative on November 2, 2024, after prolonged antibiotic treatment. Laboratory findings at admission are included (Table 1), along with evidence of bilateral septic emboli (Figure 1), degenerative changes in the lumbar spine, cholelithiasis, and hepatic steatosis. TTE and TEE showed a large vegetation on the tricuspid valve with associated thickening and regurgitation (Figure 2).

**Table 1:** Patient laboratory findings at admission.

Parameter	Normal Value	Patient Value / Finding
WBCs	4.0-11.0 × 10⁹/L	12.9 × 10⁹/L (elevated)
Platelets	150-450 × 10⁹/L	55 → 41 × 10⁹/L (markedly decreased)
Procalcitonin	<0.1 ng/mL	5.65 ng/mL (elevated; suggests bacterial sepsis)
D-dimer	<500 ng/mL	>10,000 ng/mL (markedly elevated; consistent with DIC/sepsis)
Fibrin Degradation Products	<5 µg/mL	≥20 µg/mL (elevated; consistent with DIC)
Bilirubin (Total)	0.1-1.2 mg/dL	3.7 mg/dL (elevated; possible liver involvement or hemolysis)
Alkaline Phosphatase (ALP)	44-147 U/L	238 U/L (elevated; liver or biliary origin)
Aspartate Aminotransferase (AST)	10-40 U/L	81 U/L (elevated; hepatocellular injury)
Initial Antibiotics	N/A	Vancomycin (25 mg/kg) + Ceftriaxone (2 g IV)
Definitive Therapy	Based on susceptibility	Cefazolin → transitioned to Nafcillin due to persistent sepsis
Blood Culture Status	Negative	Positive initially; turned negative on November 2, 2024
Imaging Findings	N/A	Bilateral septic emboli, lumbar spine changes, cholelithiasis, hepatic steatosis

Cardiology and Cardiothoracic Surgery were consulted for the large tricuspid valve vegetation. Surgical intervention was deemed too high risk, and the patient was managed medically with long-term IV nafcillin. Infectious Disease managed her past HIV diagnosis (CD4: 146.2) with Biktarvy and Bactrim for prophylaxis. Multisystem complications, including septic emboli to the lungs, abdomen, and joints, were treated with abscess drainage, repeat imaging, and continued IV antibiotics. Despite gradual symptomatic improvement and resolution of bacteremia, the patient experienced severe chronic pain, withdrawal symptoms requiring suboxone, and persistent emotional distress.

As the patient’s hospitalization progressed, she grew weary of continued treatment, citing poor quality of life and minimal perceived benefit. After extensive discussions regarding prognosis, the risks of treatment cessation, and the progression of infective endocarditis, she opted to transition to home hospice care. The care team ensured pain management with scheduled suboxone, initiated hospice services, and discontinued IV antibiotics per the patient’s wishes. She was discharged home against medical advice after declining to wait for formal hospice arrangements.

## Discussion

The pathophysiology of IE involves the introduction of pathogens into the bloodstream, resulting in endothelial damage. Platelets and fibrin coat the damaged endothelium, forming a sterile thrombus that becomes a scaffold for microbial colonization. IE commonly involves left-sided valves due to the increased progression of degenerative processes [2-3]. The microbiology of typical IE varies depending on the source. *Streptococcus viridans*, *Enterococcus*, and *Staphylococcus epidermidis* are associated with dental procedures, genitourinary or GI processes, and prosthetic valves or indwelling devices, respectively [3]. In our patient, repeated trauma from IVDU likely caused mechanical injury to the vascular endothelium, leading to turbulent blood flow and damage to the endothelial lining of the heart [4-5]. The tricuspid valve is the prime suspect due to its proximity to venous return. *Staphylococcus aureus *is the predominant pathogen in IE among IVDU patients, contributing to approximately 60% of cases, due to its ability to adhere to normal vascular endothelium [6]. This highlights how the unique risk factors and microbial dynamics of IVDU converge to create a distinct pathophysiological pathway for IE, with the tricuspid valve often at the center of this destructive process.

This case highlights several ethical dilemmas encountered in the care of a patient with IE and a history of IV drug use. The standard treatment for IE involves 6-8 weeks of IV, pathogen-specific antibiotic therapy, such as vancomycin or daptomycin for *Staphylococcus aureus*, penicillin G or ceftriaxone (with or without gentamicin) for streptococci, and ampicillin combined with gentamicin or ceftriaxone for enterococci, to clear the infection and prevent serious complications [7]. The patient decided to discontinue medical treatment and transition to hospice care, despite understanding the risks of worsening infection, ongoing pain, and potential death. The medical team faced a dilemma between respecting her autonomy and upholding the principle of beneficence. They had to ensure the patient possessed the mental capacity to make an informed decision about withdrawing care. This assessment was complicated by her history of substance use, intermittent pain, emotional distress, and potential cognitive impacts of infection. Ensuring that her decision was not influenced by temporary states of distress was crucial. The principle of nonmaleficence was also challenged, as withholding antibiotics and discharging her in an unstable condition could accelerate disease progression. The patient’s desire to transition to hospice care and spend the holidays at home introduced further tension between honoring her wishes and managing familial expectations. Her estranged family and friends had differing views, which the care team had to navigate while prioritizing the patient’s preferences. Finally, her history of substance use complicated palliative care planning, as effective pain management with suboxone had to be balanced against the risk of misuse or addiction. This case underscores the complexities of balancing medical, ethical, and social considerations in a patient-centered manner, respecting autonomy while ensuring informed decision-making and compassionate care.

## Conclusions

This case highlights the complexities of managing IE in a patient with a long-standing history of IVDU, underscoring the importance of prompt diagnosis, multidisciplinary collaboration, and patient-centered care. Ethical dilemmas, particularly the balance between respecting patient autonomy and ensuring beneficence, illustrate the challenges of addressing both the medical and social dimensions of care. Ultimately, the case demonstrates the critical need for compassionate communication and a deep respect for patient preferences, even when navigating difficult clinical and ethical decisions.
